# Estimating Phase Amplitude Coupling between Neural Oscillations Based on Permutation and Entropy

**DOI:** 10.3390/e23081070

**Published:** 2021-08-18

**Authors:** Liyong Yin, Fan Tian, Rui Hu, Zhaohui Li, Fuzai Yin

**Affiliations:** 1 Department of Internal Medicine, Hebei Medical University, Shijiazhuang 050011, China; qhdylywn@stu.hebmu.edu.cn; 2 School of Information Science and Engineering (School of Software), Yanshan University, Qinhuangdao 066004, China; tf@stumail.ysu.edu.cn (F.T.); hurui@stumail.ysu.edu.cn (R.H.); lizhaohui@ysu.edu.cn (Z.L.); 3 Hebei Key Laboratory of Information Transmission and Signal Processing, Yanshan University, Qinhuangdao 066004, China

**Keywords:** neuronal oscillations, phase–amplitude coupling, permutation, mutual information, entropy

## Abstract

Cross-frequency phase–amplitude coupling (PAC) plays an important role in neuronal oscillations network, reflecting the interaction between the phase of low-frequency oscillation (LFO) and amplitude of the high-frequency oscillations (HFO). Thus, we applied four methods based on permutation analysis to measure PAC, including multiscale permutation mutual information (MPMI), permutation conditional mutual information (PCMI), symbolic joint entropy (SJE), and weighted-permutation mutual information (WPMI). To verify the ability of these four algorithms, a performance test including the effects of coupling strength, signal-to-noise ratios (SNRs), and data length was evaluated by using simulation data. It was shown that the performance of SJE was similar to that of other approaches when measuring PAC strength, but the computational efficiency of SJE was the highest among all these four methods. Moreover, SJE can also accurately identify the PAC frequency range under the interference of spike noise. All in all, the results demonstrate that SJE is better for evaluating PAC between neural oscillations.

## 1. Introduction

Neuronal oscillations play a significant role in memory and learning [1,2]. In the study of cognitive processes, phase–amplitude coupling is a promising measurement [3]. Continuous electrophysiological signals which are recorded at macroscopic and mesoscopic levels, such as electroencephalogram (EEG) and local field potential (LFP), present rhythmical characteristics called neuronal oscillations [4]. Generally, they can be divided into five frequency bands, including delta (0.5–4 Hz), theta (4–8 Hz), alpha (8–12 Hz), beta (12–30 Hz), and gamma (above 30 Hz) oscillations [1,5,6]. The interaction between neuronal oscillations of different frequency bands is implemented by a communication mechanism, which is known as cross-frequency coupling (CFC) [7]. In previous experiments and studies, three types of CFC have been observed and reported, including PAC [8,9,10,11], phase–phase coupling (PPC) [12,13], and amplitude–amplitude coupling (AAC) [14,15,16].

Particularly, more reports focused on PAC because of its ubiquity in electrophysiology significance in brain functions. The PAC is generally defined as that the amplitude of a higher-frequency oscillation is modulated by the phase of a lower-frequency oscillation [17]. Moreover, the PAC has been studied in many functions, such as memory, attention selection, and sensory information detection [18,19]. It is widely observed in the hippocampus; neocortex; and basal ganglia from rats, mice, monkeys to humans [20,21,22]. Several quantitative measures have been proposed to characterize PAC, such as phase-locking value (PLV) [23], mean vector length (MVL) [24], Kullback–Liebler (KL) based modulation index (KLmi) [9], and generalized linear modeling (GLM) [25]. Overall, these different methods were proposed based on different principles and focused on different purposes, which can be found in comparative studies [9,26,27,28,29]. Recently, based on permutation entropy [30] and mutual information theory, a new method called permutation mutual information (PMI) was applied to measure PAC [31]. Although the advantage of the PMI approach has been described, it failed to account for the multiple time scales inherent in the physiological systems. To solve this problem, Aziz et al. improved the permutation entropy (PE) and called this modified procedure multiscale permutation entropy (MPE). By comparing the analysis results of MPE and PE on physiological signals, it is proved that MPE is more robust [32]. Therefore, in the present study, we proposed a novel approach named MPMI. It measures the PAC by evaluating the mutual information of multiscale permutations between two neural oscillations, which is developed from the MPE on the basis of mutual information theory.

It is well known that the classical conditional mutual information (CMI) is initially proposed to characterize the weak interaction between two phase signals with similar rhythms [33]. Cheng et al. improved it and successfully applied it in measuring PAC [34]. Afterward, Li et al. presented a new method called PCMI for assessing the directionality between two signals generated by different neuronal populations [35]. The simulations show that this method is superior to the CMI method for identifying the coupling direction. Therefore, we will use PCMI to estimate PAC. Joint entropy is a nonlinear measure used to estimate the uncertainty between one time series and another [36]. In order to weaken or eliminate the influence generated by the probability distribution of the original series, we proposed a symbolized time series to replace the original time series to calculate the probability distribution, called SJE; it can be used to measure PAC. Cui et al. proposed a novel approach to quantify the synchronization strength of EEG, which was named as normalized weighted-permutation mutual information (WPMI) [37]. It overcomes the shortcomings of the PMI algorithm. Specifically, in the extraction of the ordinal patterns, only the information of the order structure is reserved, and a large amount of amplitude information is lost. The simulation model shows that WPMI can reflect the amplitude characteristics of the time series while estimating the synchronization strength of the time series. Therefore, we will use the WPMI method to measure PAC.

Finally, we will evaluate the performance of these four algorithms (MPMI, PCMI, SJE, and WPMI) through simulation data and hope that these methods can help researchers understand the characteristics of PAC in neural oscillations and further explore the structure and function of the brain.

## 2. Materials and Methods

### 2.1. Multiscale Permutation Mutual Information

**Figure 1:**
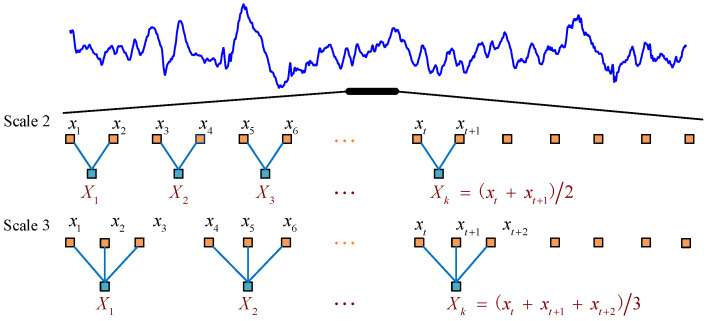
Schematic illustration of the coarse-graining procedure for scales *s* = 2 and *s* = 3.

The MPMI is developed based on MPE [32] and mutual information theory. For a time series {x(t);t=1,2,⋯,N}, we first average a successively increasing number of data points in non-overlapping windows to construct a continuous coarse-grained time series [38]. The coarse-grained time series $X_{k}^{s}$ is calculated according to the following formula:

(1) $$X_{k}^{s}=\frac{1}{s}\sum_{t=(k-1)s+1}^{ks}x(t),$$

where *s* denotes the scale factor and 1≤k≤N/s. The length of each coarse-grained time series is equal to the length of the original time series divided by the scale factor *s*. When *s* = 1, the coarse-grained time series is the original time series, and the calculated entropy value is the permutation entropy value. An example of a coarse-graining procedure is presented in Figure 1.

**Figure 2:**
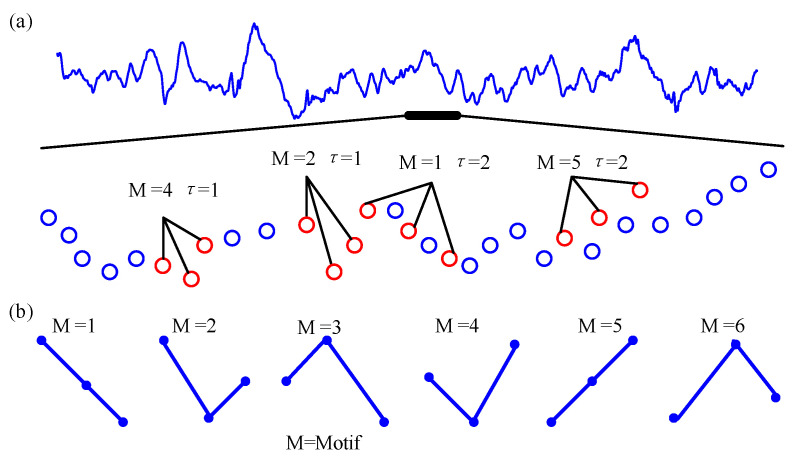
Example of sorting modes. (**a**) A segment of real EEG signal (**upper**) and some of the motifs (*m* = 3, and *τ* = 1 or 2) (**bottom**). (**b**) All of the motifs are of the order of 3 (3!). M represents the motif of the ordinal patterns.

Secondly, when the scale factor is *s*, calculate the coarse-grained time series $\left\{X_{k}^{s}\right\}$ embedding delay expression $Z_{1,l}^{m,\tau ,s}=\{X_{l}^{s},X_{l+\tau }^{s},\cdots ,X_{l+(d-1)\tau }^{s}\}$,l=1,2,3,⋯M−(m−1)τ, where *m* is the embedding dimension, and *τ* is the time delay. Then, $Z_{1,l}^{m,\tau ,s}$ is arranged in increasing order of magnitudes. When the two values of $Z_{1,l}^{m,\tau ,s}$ are equal, they are sorted according to the size of the subscript. Therefore, each of the sub-vectors in *m* dimensional space is mapped into an ordinal pattern $\pi_{i}^{m,\tau },i=1,2,\cdots m!$. Each of the T=M−(m−1)τ sub-vectors corresponds to one of m! possible arrangements. For instance, if the embedding dimension is set as 3, it will take 6 motifs for each time series, which is illustrated in Figure 2b. Figure 2a shows an example of some permutation patterns that may appear in the signal. Then, we can calculate the number of occurrences of each ordinal pattern $\pi_{i}^{m,\tau }$ which is indicated by $C_{i}$. Consequently, the probability $P(X=\pi_{i}^{m,\tau })$ of the occurrence of each symbol is obtained from the reconstructed sequence:

(2) $$P(X=\pi_{i}^{m,\tau })=\frac{C_{i}}{T},i=1,2,\cdots ,m!$$

Following, we define the probability distribution of {x(t)} as $p_{x}$, and then, the MPE of {x(t)} is defined as

(3) $$MPE(X)=-\sum_{l=1}^{m!}p_{x}\log(p_{x})$$

Consider two-time series {x(t)} and {y(t)}; let MPE(X) and MPE(Y) denote their MPE. The joint probability $p_{xy}=p(\pi_{i}^{m,\tau },\pi_{j}^{m,\tau })$ of each symbol occurrence in the signal be calculated [35]. Thus, the multiscale joint permutation entropy MPE(X,Y) can be denoted by

(4) $$MPE(X,Y)=-\sum_{i=1}^{m!}\sum_{j=1}^{m!}p_{xy}log(p_{xy})$$

Afterwards, on the basis of MPE and mutual information theory, we can define *MPMI* as follows:

(5) MPMI(X;Y)=MPE(X)+MPE(Y)−MPE(X,Y)

Finally, the normalized multiscale permutation mutual information $\left(MPMI_{Nor}\right)$ can be defined as

(6) $$MPMI_{Nor}=\frac{MPMI(X;Y)}{\min(MPE(X),MPE(Y))}$$

where the $MPMI_{Nor}$ ranges from 0 to 1. The greater the value of $MPMI_{Nor}$, the stronger the relationship between X and Y.

### 2.2. Permutation Conditional Mutual Information

Presumably, two-time series $X={\left(X_{1},X_{2},\cdots ,X_{n}\right)}^{T}$ and $Y={\left(Y_{1},Y_{2},\cdots ,Y_{n}\right)}^{T}$, based on the Shannon information theory and the permutation method introduced in Section 2.1, the PE and the joint permutation entropy of the time series can be calculated by the following formulas:

(7) $$PE(X)=-\sum_{l=1}^{m!}p_{x}ln(p_{x})$$

(8) $$PE(Y)=-\sum_{l=1}^{m!}p_{y}ln(p_{y})$$

(9) $$PE(X,Y)=-\sum_{i=1}^{m!}\sum_{j=1}^{m!}p_{xy}log(p_{xy})$$

Mathematically, the PCMI can be defined by

(10) $$PCMI_{X\to Y}^{\delta }=PCMI(X;Y_{\delta }|Y)=PE(X,Y)+PE(Y_{\delta },Y)-PE(Y)-PE(X,Y_{\delta },Y)$$

where $Y_{\delta }$ is an observable derived from the state of the process Y’s *δ* steps delay, i.e., $Y_{\delta }:y_{t+\delta }=y_{t}$. In general, the value of *δ* ranges from 3 to 15 [39]. At some later time points, the information that is transmitted from one process X to another process Y can be defined as

(11) $$PCMI_{X\to Y}=\frac{1}{N}\sum_{\delta =1}^{N}PCMI_{X\to Y}^{\delta }$$

where the *N* is the maximal later points. $PCMI_{X\to Y}$ means that the coupling strength of X to Y.

### 2.3. Symbolic Joint Entropy

Given the time series as X=[x(1),x(2),…,x(N)], sequence reconstruction is to convert X into non-overlapping sub-vectors $X_{k}$. The conversion of the new vector is shown as

(12) $$\{\begin{array}{c} X_{1}=[x(1),x(2),\cdots ,x(m)]\\ X_{2}=[x(m+1),x(m+2),\cdots ,x(2*m)]\\ \vdots \\ X_{k}=[x((k-1)*m+1),\cdots ,x(k*m)] \end{array}$$

where *m* represents the embed dimension, k=1,2,⋯,M=[N/m]. After generating $X_{k}$, we can symbolize $X_{k}$ in accordance with certain rules to reduce the requirements for the original sequence. The basic principle of the permutation is to replace the original value with anon-negative integer. For a given window, it can be denoted by a symbol according to the permutation rule in the Table 1. We take *m* = 3 as an example, as demonstrated in Table 1.

Therefore, we can get a symbolized time series $\left\{s_{x}(t);t=1,2,\cdots ,M*m\right\}$. Similarly, symbolized time series of the time series {y(t);t=1,2,⋯,N} can be expressed as $\left\{s_{y}(t);t=1,2,\cdots ,M*m\right\}$. After that, calculate the probability of occurrence of the corresponding time $\pi_{l}$ in the two symbolized time series:

(13) $$p_{l}=\frac{\#\pi_{l}}{M*m}l=1,2,\cdots ,m^{2}$$

where $\#\pi_{l}$ denotes its frequency of occurrence in the time series. $\pi_{l}$ can be expressed as

(14) $$\pi_{l}=\left\{\left[\begin{array}{c} 0\\ 0 \end{array}\right],\left[\begin{array}{c} 0\\ 1 \end{array}\right],\cdots ,\left[\begin{array}{c} 0\\ m-1 \end{array}\right],\left[\begin{array}{c} 1\\ 0 \end{array}\right],\left[\begin{array}{c} 1\\ 1 \end{array}\right],\cdots ,\left[\begin{array}{c} 1\\ m-1 \end{array}\right],\cdots ,\left[\begin{array}{c} m-1\\ 0 \end{array}\right],\cdots \left[\begin{array}{c} m-1\\ m-1 \end{array}\right]\right\}$$

Subsequently, the symbolic joint entropy is defined as

(15) $$E_{SJ}=-\sum_{\underset{p_{l}\neq 0}{l=1}}^{m^{2}}p_{l}log_{2}p_{l}$$

For two series without correlation, after symbolizing, the occurrence probability of all potential symbol vectors is approximately equal, i.e., $\frac{1}{m^{2}}$. In this case, the symbolic entropy achieves the theoretical maximum. It can be defined as

(16) $$E_{SJ}=-\sum_{l=1}^{m^{2}}\left(\frac{1}{m^{2}}log_{2}\frac{1}{m^{2}}\right)=log_{2}m^{2}$$

On the contrary, the minimum symbol joint entropy $\left(log_{2}m\right)$ is obtained when the series are completely linearly correlated. In order to minimize the influence of the parameter *m*, we further defined a normalized coupling coefficient $\left(C_{SJ}\right)$ as

(17) $$C_{SJ}=1-\frac{E_{SJ}-log_{2}m}{log_{2}m^{2}-log_{2}m}=2-\frac{E_{SJ}}{log_{2}m}$$

The index of $C_{SJ}$ is positively correlated with the coupling strength between the two series. The value 1 indicated the perfect inter-series coupling whereas the value 0 suggested no inter-series correlation.

### 2.4. Weighted-Permutation Mutual Information

Given two time series *X* and *Y* which are embedded into *m* dimensional space, each of the sub-vectors is assigned to an ordinal pattern $\pi_{i}$. Specifically, the weighted probability $p_{\omega }$ for each ordinal pattern of *X* can be calculated as

(18) $$p_{\omega }(\pi_{i})=\frac{\sum I_{\pi_{i}}(X_{i})\omega_{i}}{\sum I_{\Pi }(X_{i})\omega_{i}}$$

where Π is a collection of ordinal patterns $\pi_{i},i=1,2,\cdots ,m!$. $I_{\pi_{i}}(X_{i})=1$, if the sub-vector $X_{i}$ can be mapped to ordinal pattern $\pi_{i}$, otherwise $I_{\pi_{i}}(X_{i})=0$. $\omega_{i}$ is a weight which is determined by a specific feature of each sub-vector $X_{i}$. Different features are selected according to the needs, but the relationship $\sum_{i}p_{w}(\pi_{i})=1$ is always present. The weight $\omega_{i}$ is calculated by

(19) $$\omega_{i}=\frac{1}{m}\sum_{k=1}^{m}{\left(x_{i+(k-1)\tau }-{\bar{X}}_{i}\right)}^{2}$$

where ${\bar{X}}_{i}$ is mean of sub-vector $X_{i}$ obtained by the following formula:

(20) $${\bar{X}}_{i}=\frac{1}{m}\sum_{k=1}^{m}x_{i+(k-1)\tau }$$

Therefore, the probability distribution $p_{\omega x}$ of weighted ordinal pattern in time series *X* is obtained. $p_{\omega y}$ can be calculated in the same way.

For these two-time series, $\pi_{i}$ and $\pi_{j}$ are used to indicate the ordinal patterns of $X_{k}$ and $Y_{k}$, respectively. Thus, m!∗m! joint ordinal patterns are available. A new method for calculating the probability of each joint ordinal pattern is proposed as follows:

(21) $$p_{\omega }(\pi_{ij})=\frac{\sum I_{\pi_{ij}}(X_{i};Y_{j})\omega_{ij}}{\sum I_{\Pi }(X_{i};Y_{j})\omega_{ij}}$$

where Π is a collection of ordinal patterns $\pi_{ij}$.$I_{\pi_{ij}}(X_{i};Y_{j})=1$, if the sub-vector $X_{i}$ and $Y_{j}$ can be mapped into ordinal pattern $\pi_{ij}$, otherwise $I_{\pi_{ij}}(X_{i};Y_{j})=0$. Weight $\omega_{ij}=\omega_{i}\omega_{j}$, $\omega_{i}$ and $\omega_{j}$ denote the weight of the sub-vector $X_{i}$ and $Y_{j}$, respectively. Thus, the weighted probability distribution of joint ordinal patterns $p_{wxy}$ is obtained. According to the information theory, the WPE of the time series *X* and *Y* can be calculated as

(22) $$WPE(X)=-\sum_{i=1}^{m!}p_{\omega x}log(p_{\omega x})$$

(23) $$WPE(Y)=-\sum_{j=1}^{m!}p_{\omega y}log(p_{\omega y})$$

(24) $$WPE(X,Y)=-\sum_{i=1}^{m!}\sum_{j=1}^{m!}p_{\omega xy}\log(p_{\omega xy})$$

Then, WPMI is defined as

(25) WPMI(X;Y)=WPE(X)+WPE(Y)−WPE(X,Y).

Afterward, the WPMI can be normalized as

(26) $$WPMI_{Nor}=\frac{WPMI(X;Y)}{\max\{WPMI(X;X),WPMI(Y;Y)\}},$$

where $WPMI_{Nor}$ varies from 0 to 1. If *X* and *Y* are two perfect synchronous time series, $WPMI_{Nor}$ equals 1; if there is completely no synchronization between *X* and *Y*, $WPMI_{Nor}$ is close to 0. The stronger the synchronization between *X* and *Y*, the higher the $WPMI_{Nor}$.

### 2.5. Acquisition of Simulation Data

First, multiple sine waves with different frequencies, amplitudes, and phases are added together to generate the simulation data [40,41]. The low-frequency oscillation (LFO) and high-frequency oscillation (HFO) are denoted by low(t) and high(t), respectively. The low(t) is a 4–8 Hz theta oscillation and the high(t) is a 60–70 Hz gamma oscillation, and the sampling frequency is 1000 Hz. The amplitude of the components was inversely proportional to their frequencies. Both oscillations are normalized to a range of −1 to 1; the purpose is to eliminate the interference caused by amplitude fluctuation and maintain the fluctuation of its instantaneous frequency. The phases were randomly selected from [−π,π]. After that, the Von Mises coupling method is used to generate the amplitude series of HFO [42]:

(27) $$A_{high}(t)=\frac{c}{\exp\lambda }\exp[\lambda \times low(t)].$$

The parameter *c* controls the maximum amplitude of HFO. *λ* is a concentration parameter. The *λ* with bigger value generates the larger amplitude of HFO around the preferred phase. Specifically, the *λ* with a zero value caused the equal amplitudes of the HFO at all phases. In the following simulation analysis, parameters are *c* = 1, *λ =* 1. Consequently, the raw data with PAC can be generated as below:

(28) $$Raw=low(t)+A_{high}(t)\times high(t).$$

**Figure 3:**
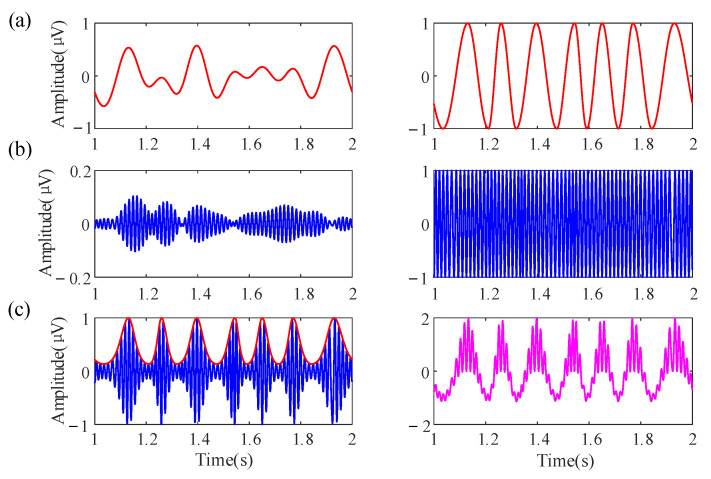
An example for generating the simulation data. (**a**) A nonstationary slow oscillation (4–8Hz, red curve, **left**), normalized to −1 to 1 (**right**), aiming to remove amplitude fluctuations and retain the fluctuation of its instantaneous frequency. (**b**) A nonstationary fast oscillation (60–70Hz, blue curve, **left**), normalized to −1 to 1 (**right**). (**c**) The amplitude envelope of the fast oscillation (red, **left**). The raw data (magenta, **right**) were the sum of the normalized slow oscillation and fast oscillation signal.

A construction example of the simulation data is shown in Figure 3.

Afterward, to regulate the PAC strength, the raw data are combined with an interferential signal (IFS) and normalize in the same way. Therefore, the original signal can be reconstructed as

(29) $$Raw^{\prime }=k\times low(t)+(1-k)\times IFS(t)+A_{high}(t)\times high(t),$$

**Figure 4:**
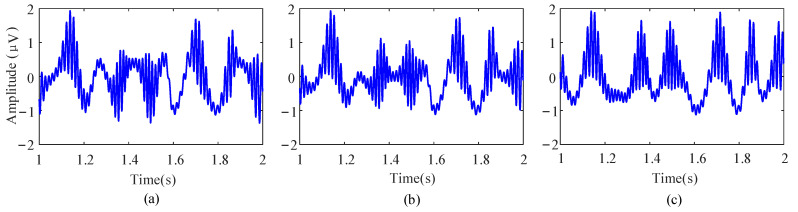
Examples of the simulation data with different PAC strengths. (**a**) *k* = 0.3. (**b**) *k* = 0.5. (**c**) *k* = 0.8.

The parameter *k* represents the coupling strength, ranging from 0 to 1. Figure 4 shows the simulation results under different coupling strengths.

### 2.6. Calculating Phase–Amplitude Coupling

The coupling strength between the amplitude $A_{high}(t)$ of HFO and phase $\theta_{low}(t)$ of LFO can be measured by MPMI, PCMI, SJE, and WPMI methods. However, the phase series $\theta_{low}(t)$ of the low(t) is a periodic function with monotonically increase in each cycle. Thus, the ordinal patterns of $\theta_{low}(t)$ would be very simple, and it is inappropriate for analyzing the coupling between $\theta_{low}(t)$ and $A_{high}(t)$.Conversely, if there is a prominent PAC between them, the magnitude orders of $\cos(\theta_{low}(t))$ will be similar to that of $A_{high}(t)$. Correspondingly, it would be beneficial for calculating PAC between $\cos(\theta_{low}(t))$ and $A_{high}(t)$.

First of all, by using Hilbert transformation:

(30) low(t)=Re(Alow(t)∗eiθlow(t)),

where $\theta_{low}(t)$ and $A_{low}(t)$ are the phase and amplitude series of the slow oscillation, respectively. Thus, $\cos(\theta_{low}(t))$ can be generated by dividing low(t) by its instantaneous amplitude $A_{low}(t)$:

(31) low(t)Alow(t)=Re(eiθlow(t))=cos(θlow(t)),

Therefore, the amplitude of $\cos(\theta_{low}(t))$ is only dependent on the phase time series of the slow oscillation, which is more conducive to the measurement of PAC.

Finally, after generating the phase time series $\cos(\theta_{low}(t))$, we further use the Hilbert transform to produce the amplitude $A_{high}(t)$ of the fast wave oscillation high(t). Then, the PAC of two time series can be obtained by $\cos(\theta_{low}(t))$ and $A_{high}(t)$.

### 2.7. Parameter Choices in the Algorithm

**Figure 5:**
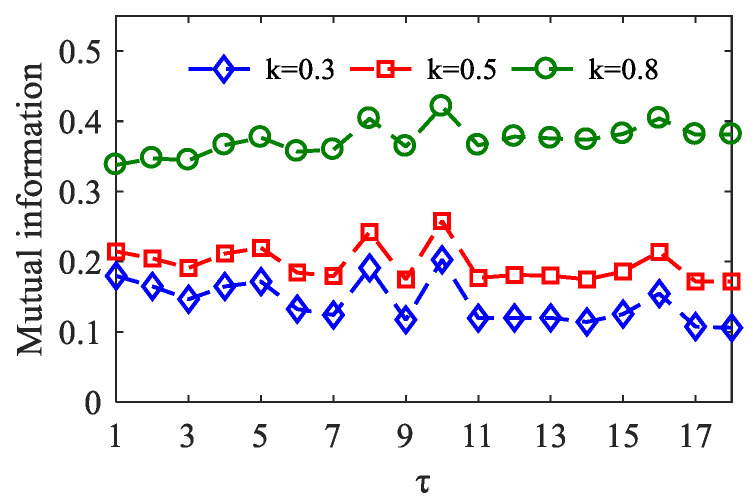
With the coupling strength *k* = 0.3, *k* = 0.5, *k* = 0.8, and *τ* ranging from 1 to 18, the curves of mutual information are between the amplitude of the HFO and the phase of the LFO. There were 100 realizations for each coupling strength.

In each algorithm, two key parameters embed dimension *m* and time lag *τ* are included. The order *m* represents the number of data points involved in the motif. Bandt et al. suggested that the value range of *m* is 3–7 [30], we set *m* = 3. The lag is referred to as the number of sample points spanned by each motif. Regarding the choice of *τ*, we refer to the mutual information method proposed by Li et al. [35]. In the simulation data, by comparing the mutual information derived from the different *τ* values, the lag *τ* corresponding to the maximal value of mutual information is an optimum parameter. As shown in Figure 5, when *τ* = 10, the mutual information reaches the maximum value between $\cos(\theta_{low}(t))$ and $A_{high}(t)$ with coupling strength *k* = 0.3, *k* = 0.5, and *k* = 0.8.

### 2.8. Parameter Choice in MPMI

**Figure 6:**
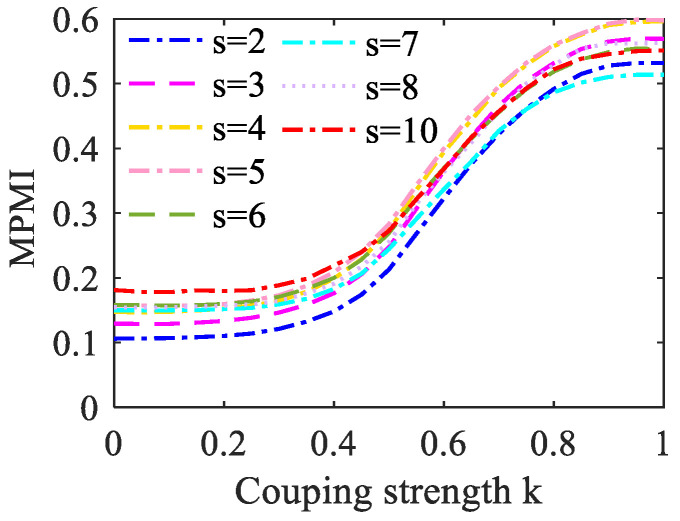
The curve of the change of MPMI value with the coupling strength *k* under different scale factors *s*. There were 100 realizations for each coupling strength.

When analyzing the MPE, the coarse-grained process is very important. The specific operation is to truncate the raw time series into several small sequences with the same length and average each segment to obtain a new time series. Therefore, the analysis of signal complexity needs to consider the choice of the scale factor *s* in the coarse-graining process. The adjacent elements of the original time series contain sequence information. If the value of the scale factor *s* is too small, the information of the relevant fragments cannot be extracted to the greatest extent, so the analysis effect is not obvious. However, when the complexity difference between the signals is small, the scale factor *s* should not be selected too large, otherwise, the difference between the signals may be eliminated in the averaging process. In this study, the MPMI value varies with coupling strength under different scale factors *s* is set. As shown in Figure 6, we can observe that MPMI is proportional to *k*. When *s* increases, the MPMI curve moves upward as a whole, but the overall change trend remains unchanged. When *k* = 0, to avoid a higher MPMI value, *s* = 3 is selected.

### 2.9. Parameter Choice in PCMI

**Figure 7:**
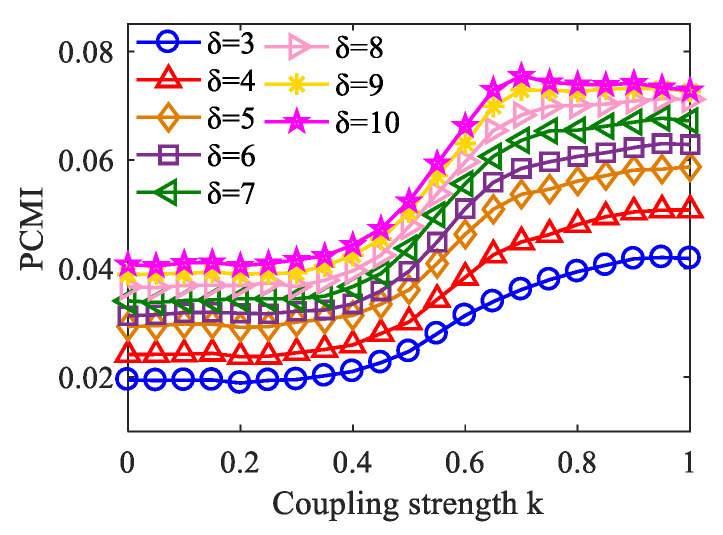
When *δ* takes different values, the PCMI curve varies with the coupling strength *k* (100 realizations for each strength).

When applying the PCMI method, it is necessary to consider the selection of parameter *δ* because of its influence on the PCMI estimation. The algorithm itself determines that the value of *δ* cannot be less than the order *m* in PCMI [33]. In the simulation data, we plotted the variation curve of PCMI with the coupling strength *k* when *δ* takes from 3 to 10. As shown in Figure 7, we can see that with the increase of *δ*, the changing trend of the PCMI curve remains constant, and increases with *k*. However, when *δ* = 10, *k* = 0.7 to 1, the PCMI value dropped, causing inaccurate measurement results. That is, when *δ* takes from 3 to 9, the PCMI estimation is stable. Therefore, in this paper, we suggest that the choice of *δ* = 5 is an appropriate option.

## 3. Results

### 3.1. Dependence on Data Length

**Figure 8:**
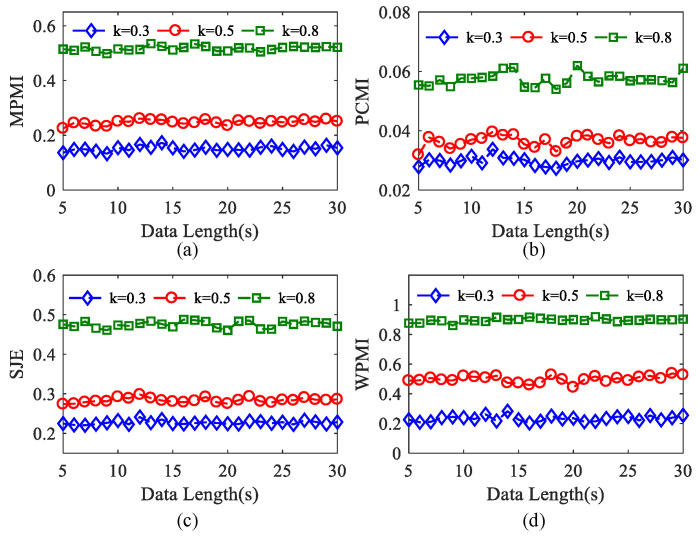
Effect of data length on the four methods at different coupling strengths (100 realizations for each strength). (**a**) MPMI. (**b**) PCMI. (**c**) SJE. (**d**) WPMI.

In order to analyze the influence of data length on four kinds of algorithms, simulated time series with data length from 5 s to 30 s (5000 to 30,000 sample points) are generated by using the simulation data. We conducted 100 experiments on each epoch set of data length when *k* = 0.3, 0.5, 0.8. It can be seen from Figure 8a,c,d that MPMI, SJE, and WPMI all present three small fluctuation horizontal curves, indicating that these three algorithms are not sensitive to changes in the data length. In addition, it can also be explained that when the PAC intensity is constant within a period, the data length has almost no effect on the performance of these three algorithms. When *k* = 0.8, compared with MPMI and SJE, the WPMI curve is more stable, and the performance is the best. As demonstrated in Figure 8b, when *k* = 0.3, 0.5, 0.8, the three curves show small fluctuations, which can reflect the PAC strength effectively. In short, these four algorithms can reflect the PAC value under different *k* within 5 s to 30 s.

### 3.2. The Effects of Coupling Coefficient on Methods

**Figure 9:**
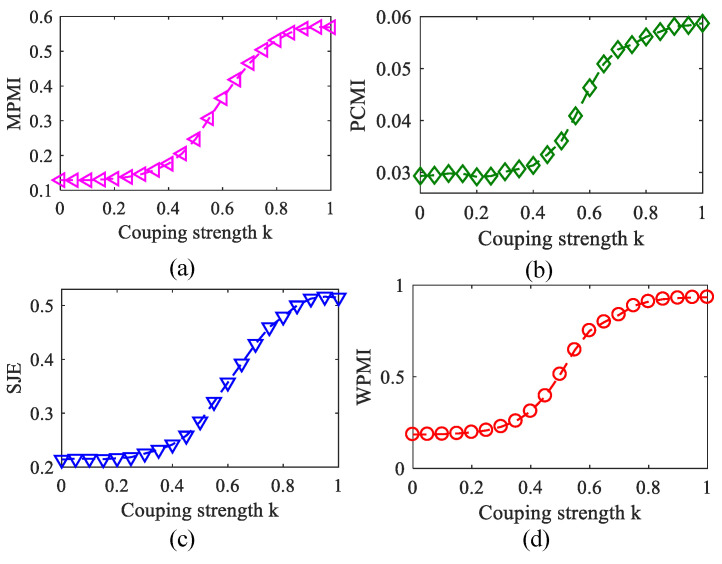
Variation of the four methods with the coupling strength (100 realizations). (**a**) MPMI. (**b**) PCMI. (**c**) SJE. (**d**) WPMI.

To analyze MPMI, PCMI, SJE, and WPMI characteristics with the changing of coupling strength, coupling coefficient *k* gradually increased from 0 to 1 with the step of 0.05. Figure 9 plots the relationship between MPMI/PCMI/SJE/WPMI and coupling strength *k*, where the PAC estimators all gradually increase as the *k* increases. However, when 0≤k≤0.2 or 0.8≤k≤1, the PAC estimators increased comparatively slowly. It shows that when the coupling is weak or strong, it is difficult to distinguish any difference of PAC intensities.

### 3.3. The Effects of Noise on Methods

**Figure 10:**
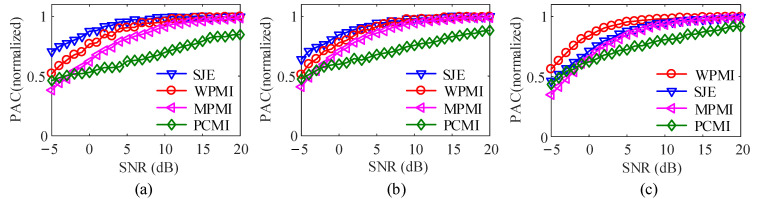
Effect of white Gaussian noise (100 realizations for each strength). (**a**) *k* = 0.3. (**b**) *k* = 0.5. (**c**) *k* = 0.8.

To analyze the effects of noise on four methods, white Gaussian noise with an SNR varying from −5 dB to 20 dB in steps of 1 dB is added to the coupled signal. The data length is 10,000. In the case of *k* = 0.3, 0.5, 0.8, respectively, Figure 10 plots the variation of PAC with white Gaussian noise and coupling strengths of *k* = 0.3, 0.5, 0.8. In order to compare the four methods in the same coordinate system, the normalization curve is realized by dividing the coupling value with noise by the coupling value without noise.

It could be seen that in Figure 10a, when *k* = 0.3, SNR from −5 dB to 6 dB, compared with the other three methods, SJE method is closer to the coupling value when there is no noise, and the ability to suppress noise better. When the value of SNR ranges from 10 dB to 20 dB, SJE, WPMI and MPMI are all close to the noise-free coupling value, while the PCMI value increases with the increase of the SNR, until SNR = 20 dB, it still does not reach the coupling value under noise-free conditions value. Figure 10b shows that when *k* = 0.5 and SNR range from is −5 dB to 5 dB, the anti-noise performance of SJE is slightly better than the other three methods. In Figure 10c, when *k* = 0.8, SNR ranges from −5 dB to 10 dB, WPMI against noise is the best, exceeding SJE. This is because under the same data length, the higher the coupling strength, the better the performance of the WPMI. Moreover, when SNR is from 7 dB to 20 dB, SJE and MPMI have similar performance.

### 3.4. Detection of the Frequency Pairs with PAC

In the process of collecting EEG data, there will inevitably be some noise interference. Therefore, in this paper, white Gaussian noise is used to simulate measurement noise [43]. In addition, when analyzing EEG signals collected by humans or animals under different behaviors and cognitive situations, we do not know the magnitude of the PAC strength value and the coupling frequency range of each stage in advance. Therefore, it is necessary to measure the PAC to obtain the best possible frequency estimation of the phase and amplitude coupling components. The PAC value can also be used to evaluate the degree of PAC affected under different behaviors or physiological and pathological mechanisms.

At present, several methods of exploring PAC have been adopted. Among them, the most popular analysis is the comodulogram, in which a “coupling palette” is computed and used to indicate the strength of the modulation of amplitude by phase within wide ranges of phase-giving and amplitude-giving frequencies, [21,44,45]. Its advantage is that the experimenter can directly observe the frequency range showing the phase and amplitude of strong coupling. However, before the analysis, the possible coupling frequency range must be chosen. We used a comodulogram to test whether these four algorithms can detect PAC frequency pairs in a given frequency range. In order to verify the accuracy of the algorithm, the frequency range of the simulation data was expanded to 1–100 Hz, and the actual PAC coupling frequency range was 4–8 Hz and 60–70 Hz. Usually, the abscissa represents the frequencies analyzed as phase-modulating $f_{P}$, whereas amplitude-modulated $f_{A}$ is represented in the ordinate axis; that is, jet colors in a given coordinated (*x*, *y*) of the bi-dimensional map indicate that the phase of the *x* frequency modulates the amplitude of the *y* frequency.

**Figure 11:**
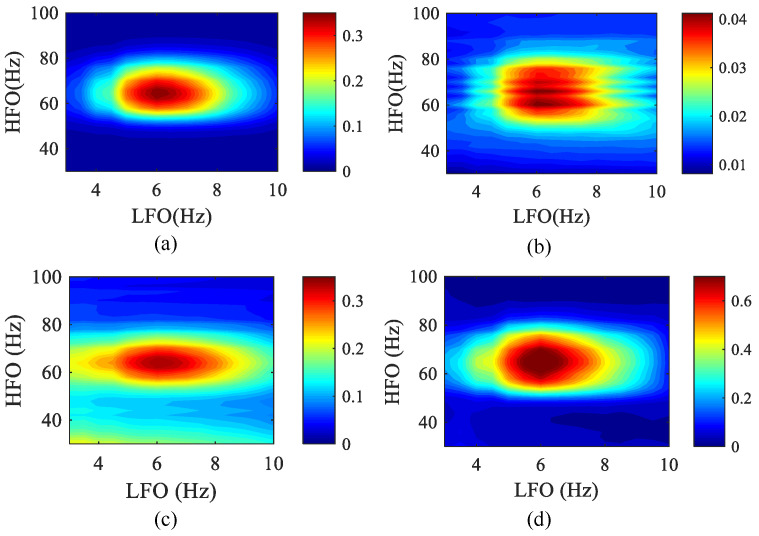
The phase–amplitude comodulograms of the four methods when white Gaussian noise is added to the simulated data (50 realizations). The abscissa represents the frequency for the modulated phase, and the ordinate represents the frequency for the modulated amplitude. The center frequencies (LFO: 3–10 Hz with a step of 0.5 Hz; HFO: 30–100 Hz with a step of 2 Hz) of the bandpass filter corresponding to the coordinates in each comodulogram. (**a**) MPMI. (**b**) PCMI. (**c**) SJE. (**d**) WPMI.

In Figure 11, we show an example of applying a comodulogram to simulate data. The comodulograms were constructed by using $f_{P}$ calculated in 0.5 Hz steps with 4 Hz bandwidths and $f_{A}$ calculated in 2 Hz steps with 10 Hz bandwidths. Next, in order to simulate the measurement noise in the EEG signal more realistically, white Gaussian noise with SNR = 5 dB was added to the original data, and the PAC comodulogram of the four methods at *k* = 0.8 was obtained as shown below. The comodulograms of MPMI and WPMI are relatively clustered and can correctly show the phase–amplitude frequency range. Although SJE is more concentrated than the MPMI and WPMI, white Gaussian noise has an impact on it. The comodulogram obtained by PCMI is relatively scattered, the frequency range of the HFO becomes wider, and white Gaussian noise has a greater impact on it. Furthermore, the running time of the four algorithms was also compared. The SJE algorithm has the fastest running time, WPMI takes the longest time, and MPMI is slightly faster than PCMI.

### 3.5. Sensibility to Spurious Coupling

The instantaneous sharp edges in the time domain correspond to the broadband harmonic components in the frequency domain. They affect the phase of low-frequency components and the amplitude of high-frequency components. Therefore, even if there is no real physiological interaction, spurious CFC may be generated [46,47,48]. In the previous simulation analysis, we only considered the interference caused by measurement noise. In fact, in real EEG recordings, noise components may be more complex and intense, such as sharp waveforms and large artifacts. Using simulated data, Kramer and colleagues believe that in some cases, sharp edges in the data may result in coupling in a wide range of frequencies-for-amplitude [49]. However, this type of coupling has not been confirmed in real the scalp or intracranial EEG data until recently, and it is unclear under what circumstances, if at all, such a situation may arise [47,50]. In this study, the Hilbert transform is used to extract the analytic signal with the instantaneous amplitude and phase components. Therefore, it inevitably leads to non-meaningful amplitude estimates for PAC computation.

**Figure 12:**
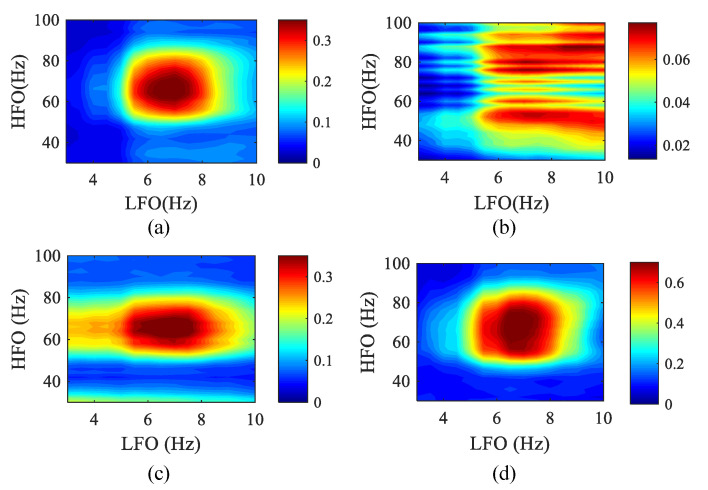
The phase–amplitude comodulograms of four methods when a train of spikes are superimposed on the background signal (50 realizations). The abscissa represents the frequency for the modulated phase, and the ordinate represents the frequency for the modulated amplitude. The center frequencies (LFO: 3–10 Hz with a step of 0.5 Hz; HFO: 30–100 Hz with a step of 2 Hz) of the bandpass filter corresponding to the coordinates in each comodulogram. (**a**) MPMI. (**b**) PCMI. (**c**) SJE. (**d**) WPMI.

Currently, existing papers have studied the detection of spurious coupling phenomena in signals without interaction [49]. Thus, we studied the impact of spike noise on the detection of PAC frequency bands by these four algorithms in the case of strong coupling. In the simulation data, set *k* = 0.8, the data length is 10 s, and a train of a spike (3 standard deviations height, 20 ms width at half-maximum, 100 ms inter-peak interval) are superimposed on the background signal. In the presence of spurious coupling, the phase–amplitude comodulograms of the four methods are obtained, as shown in Figure 12.

We can observe that compared with other methods, the SJE has a higher degree of aggregation and can better reflect the true coupling frequency range, and spurious coupling has less influence on it. In the MPMI and WPMI methods, the range of high-frequency rhythms in their comodulograms becomes wider due to the influence of spurious coupling. PCMI loses the ability to detect the coupling frequency range in the presence of spike noise.

## 4. Conclusions

In this study, the methods based on permutation analysis were applied to measure the PAC strength. The comparison was carefully performed between the performances of SJE and that of MPMI, PCMI, and WPMI. The results demonstrated that compared with other methods, the SJE algorithm exhibited superior performance, including the lower complexity and the highest computational efficiency. Furthermore, the results also show that SJE can better resist additive white Gaussian noise except for high coupling strengths where WPMI is more effective. In addition, we also found that the SJE has the ability to detect the pairs of coupling frequency in a wide frequency range when the signal is mixed with noise of low signal-to-noise ratio. moreover, it is capable to depict the comodulogram of a signal containing spike noise. In conclusion, it suggests that SJE is possibly a better choice to evaluate the PAC under certain conditions.

## Figures and Tables

**Table 1:** The permutation rule in symbolic joint entropy.

Permutation	Symbol
$x_{i}\leq x_{i+1}\leq x_{i+2}$	012
$x_{i}\leq x_{i+2}\leq x_{i+1}$	021
$x_{i+1}\leq x_{i}\leq x_{i+2}$	102
$x_{i+1}\leq x_{i+2}\leq x_{i}$	120
$x_{i+2}\leq x_{i+1}\leq x_{i}$	210
$x_{i+2}\leq x_{i}\leq x_{i+1}$	201

## Data Availability

The data presented in this study are available on request from the corresponding author.
